# Successful Treatment of Type B Insulin Resistance With Rituximab

**DOI:** 10.1210/jc.2014-3552

**Published:** 2015-02-12

**Authors:** Emmanouil-Dimitrios Manikas, Iona Isaac, Robert K. Semple, Rana Malek, Dagmar Führer, Lars C. Moeller

**Affiliations:** University Duisburg-Essen (E.-D.M., D.F., L.C.M.), Department of Endocrinology and Metabolism and Division of Laboratory Research, 45147 Essen, Germany; Wellcome Trust-Medical Research Council Institute of Metabolic Science (I.I., R.K.S.), Addenbrooke's Hospital, Cambridge CB2 0QQ, United Kingdom; and University of Maryland School of Medicine (R.M.), Division of Endocrinology, Diabetes, and Nutrition, Baltimore, Maryland 21201

## Abstract

**Context:**

Type B insulin resistance is a very rare disease caused by autoantibodies against the insulin receptor. The mortality of type B insulin resistance is high (>50%), and management of this disease is not yet standardized. We report the successful treatment of a patient with type B insulin resistance with rituximab, cyclophosphamide, and prednisone.

**Case Description:**

A 45-year-old woman presented with unintended weight loss of 20 kg, unusually widespread acanthosis nigricans, and glucose levels > 500 mg/dL, which could not be controlled with up to 600 IU/d of insulin. Because of the severity of the insulin resistance combined with features of insulin deficiency, type B insulin resistance was suspected. Detection of high levels of insulin receptor autoantibodies confirmed the diagnosis. Neither immunosuppressive therapy with Ig iv nor plasmapheresis had an effect on glucose levels or insulin dose. Because the patient's condition was deteriorating, we started rituximab (750 mg/m^2^ in two doses 2 wk apart) together with cyclophosphamide (100 mg/d orally) and dexamethasone 40 mg/d for 4 days. Two months after initiation of rituximab therapy, fasting glucose levels ranged from 80 to 110 mg/dL and could be controlled with very low insulin doses. Glycated hemoglobin decreased from 11.8 to 6.5%. Two months later, insulin therapy was stopped, and the patient showed normal blood glucose readings.

**Conclusion:**

In this patient with type B insulin resistance, Ig treatment and plasmapheresis failed to improve the condition. Finally, treatment with rituximab, cyclophosphamide, and steroids was successful in inducing a complete remission.

The syndrome of type B insulin resistance is caused by circulating autoantibodies against the insulin receptor. The manifestation occurs mainly in the fourth to sixth decade of life with female preponderance and is commonly associated with other autoimmune conditions, eg, systemic lupus erythematosus. Clinically, the condition presents with widespread acanthosis nigricans, often with severe insulin resistance, and less often with hyperandrogenism and hirsutism (1). Acanthosis nigricans tends to improve with the disappearance of circulating antibodies (2). The syndrome is caused by polyclonal antibodies (typically IgG) against the insulin receptor that lead to either insulin resistance or fasting hypoglycemia, depending on the blocking or stimulating activity of the antibodies and their titers. Mortality of type B insulin resistance is high (>50% within 10 y) (2). Therapeutic approaches such as insulin sensitization with metformin and thiazolidinediones, immunomodulating agents (corticosteroids, cyclophosphamide, cyclosporine A, azathioprine), plasmapheresis, or combinations of the above have shown mixed results (2–8), and treatment is not yet standardized. In 2010, a group at the National Institutes of Health (NIH) published the largest case series in which a new treatment protocol with rituximab, a B-cell-depleting monoclonal anti-CD20 antibody, was tested in their patient population (6). To date, this has not been validated in other patients outside of the NIH.

## Case Report

**Figure 1.**
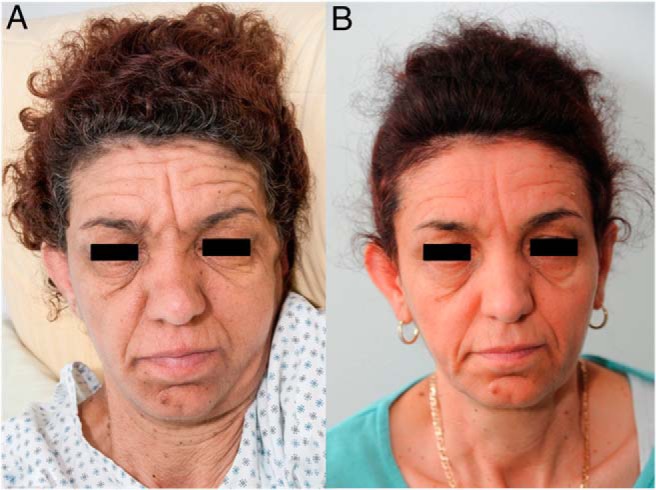
A 45-year-old female patient with acanthosis nigricans due to type B insulin resistance at diagnosis (A) and 4 months after rituximab treatment (B).

A 45-year-old Caucasian woman presented with weight loss of 20 kg over 9 months and acanthosis nigricans of her face and lumbar and groin areas (Figure 1A). One year earlier, diabetes mellitus had been diagnosed. The initial treatment with metformin and sitagliptin was unsuccessful. Plasma glucose levels (500 mg/dl) and glycated hemoglobin (HbA1c, 11.3%) were high. Intensive conventional insulin therapy and administration of 600 IU/d via insulin pump failed to achieve acceptable blood glucose levels.

At admission, her body mass index was only 18 kg/m^2^. We initiated continuous iv insulin. To achieve blood glucose levels of approximately 300 mg/dL, approximately 6 IU/h were required. After administering insulin iv for 72 hours, we started an intensive conventional insulin therapy plan (isophan insulin [NPH; Protaphane, Novo Nordisk Pharma GmbH] 50–50–50 IU, Insulin human rDNS [NovoRapid, Novo Nordisk Pharma GmbH] 26–34–34 IU, plus correction with a factor of 1:15, with a blood glucose target of 90–120 mg/dL).

**Figure 2.**
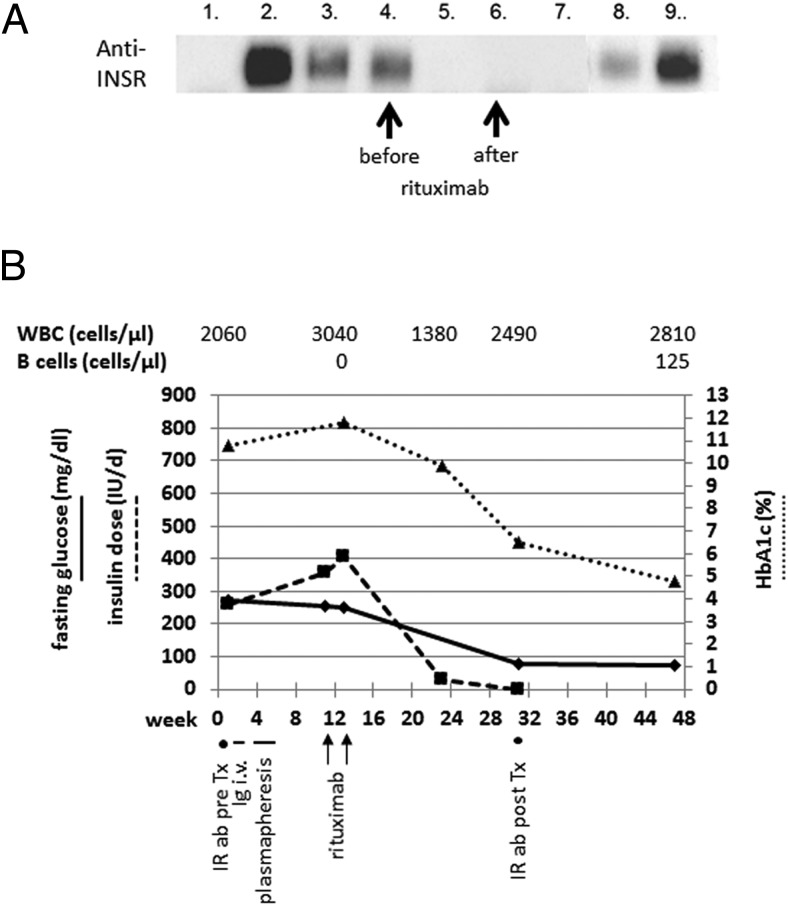
A, Anti-INSR autoantibody assay for our patient (P1070) before and after treatment (25-min exposure). Lane 1, Negative control serum (2 μL), negative for anti-INSR autoantibody; lane 2, positive control serum (2 μL), positive for anti-INSR autoantibody; lane 3, positive control serum (0.2 μL), positive for anti-INSR autoantibody; lane 4, P1070 serum before therapy (2 μL); lane 5, P1070 before therapy (0.2 μL); lane 6, P1070 after therapy (2 μL); lane 7, P1070 after therapy (0.2 μL); lane 8, cell lysate containing insulin receptor 1:3 dilution; and lane 9, cell lysate containing insulin receptor. INSR, insulin-receptor. B, Time line (weeks) for fasting glucose (mg/dL; solid line), daily insulin dose (IU/d; dashed line), and HbA1c (%; dotted line) as well as white blood cell (WBC) and B cell count. The arrows indicate application of rituximab/cyclophosphamide/prednisone, the short lines indicate the Ig treatment and plasmapheresis, and the dots indicate timing of insulin receptor antibody tests. ab, antibodies; Ig, immunoglobulins; IR, insulin-receptor; Tx, treatment.

Extensive examination failed to reveal any (para)neoplastic cause for the weight loss and insulin resistance. We considered the possibility of type B insulin resistance syndrome because of acanthosis nigricans combined with weight loss and elevated serum markers of autoimmunity, especially anti-Sjögren's-syndrome-related antigen A and antiribosomal P protein (Supplemental Table 1). However, an initial insulin receptor antibody assay was negative. Finally, an immunoprecipitation assay was strongly positive for anti-insulin-receptor antibodies (Figure 2A), confirming the diagnosis of type B insulin resistance.

Neither Ig iv (Intratect 20 g/d; Biotest Pharma GmbH) over 6 days nor plasmapheresis (five times in 14 d) improved her blood glucose or allowed reduction of the daily insulin dose. We therefore started the patient on a combination protocol of rituximab (750 mg/m^2^ in two doses 2 wk apart), cyclophosphamide (100 mg/d orally, continuously), and dexamethasone (40 mg/d for 4 days every month), in accordance with the NIH protocol (Figure 2B) (6), which was well tolerated. B cells were depleted already 2 weeks after the first rituximab application but returned to almost normal levels 4 months later without relapse (Figure 2B). Cyclophosphamide was temporarily withdrawn due to low white blood cells. No other major side effects were reported. Over the next 2 months, her daily insulin doses could be reduced to 30 IU/d, already indicating a response to therapy. The patient's well-being greatly improved. Fasting glucose levels ranged from 80 to 110 mg/dL, and the HbA1c decreased from 11.8 to 9.9%. Four months after her initial rituximab dose, insulin treatment could be withdrawn completely, and blood glucose levels remained within the normal range from 66 to 107 mg/dL. HbA1c continued to decrease to 6.5% (Figure 2B). The acanthosis nigricans improved (Figure 1B). Congruent with the complete clinical remission, insulin receptor autoantibodies were now negative (Figure 2A). We therefore discontinued cyclophosphamide and dexamethasone and started her on a maintenance regime with azathioprine 100 mg daily for 1 year. Azathioprine was chosen because of the experience with this immunosuppressive drug in systemic lupus erythematosus and because many type B insulin resistance patients are positive for lupus-associated antibodies, including our patient. She has been in remission since then (Figure 2B).

## Discussion

We report a patient with extreme insulin resistance with features of insulin deficiency due to insulin receptor autoantibodies. Treatment of type B insulin resistance is challenging. Its prevalence is unknown but is very low, precluding prospective and controlled trials. We initially tried plasmapheresis and Ig iv, expecting that this would eliminate the anti-insulin receptor autoantibodies rapidly. Yet, blood glucose levels and insulin requirements did not respond. We therefore tried a previously published protocol including rituximab, cyclophosphamide, and pulse corticosteroids aiming to control antibody-producing B lymphocytes and to suppress the activity of pre-existing antibody-producing plasma cells. An initial case report of a patient with type B insulin resistance treated with rituximab was published in 2004 (9). In 2010, Malek et al (6) published the largest case series using a standardized protocol for type B insulin resistance in seven patients. Our 45-year-old female Caucasian patient resembles these patients (five females, two males; average age, 41.7 y; range, 17–64 y), but she has a different ethnic background (six African American, one First Nation Canadian). All initially reported patients were positive for antinuclear antibodies; three had systemic lupus erythematosus, and one had mixed connective tissue disease. Our patient also was antinuclear antibody-positive, especially for lupus-associated antibodies (see Supplementa1 Table 1), but without cutaneous signs of lupus erythematosus. Her metabolic parameters, fasting glucose and HbA1c, were similar to the hyperglycemic NIH patients, but her insulin dose shortly before rituximab treatment was relatively low at 360 IU/d, compared to 750 to 18 000 (average 5000) IU/d in the NIH patients. Relatively less severe insulin resistance may explain why our patient reached complete remission within 4 months after one treatment cycle, whereas the NIH patients required on average 1.6 cycles (range, 1–2.5) and reached remission within 8 months (range, 2.5–27).

Use of rituximab in our patient was off label, but this was well justified by the data from the NIH patients, the severity of the disease with a mortality rate > 50%, and the lack of alternative treatments for a patient who was clinically deteriorating. This view was shared by the patient's health insurance provider, and the costs of this off-label treatment were covered by insurance.

Recently, we learned of the success of this treatment regime in a patient from Ecuador (10). Therefore, in addition to the mostly African American patients of the NIH series, success of this treatment regime could be confirmed in patients from Europe and from Latin America.

## Conclusion

Type B insulin resistance is a rare disease with high mortality. Its therapy is not yet standardized. A treatment regime with rituximab, cyclophosphamide, and steroids led to complete remission in all nine patients known to be treated with this regime so far, which suggests that this protocol should be used as first-line treatment.
